# Connexin43 Expression Increases in the Epithelium and Stroma along the Colonic Neoplastic Progression Pathway: Implications for Its Oncogenic Role

**DOI:** 10.1155/2011/561719

**Published:** 2011-06-30

**Authors:** Yusheng Han, Paul J. Zhang, Terina Chen, Sabrina W. Yum, Teresa Pasha, Emma E. Furth

**Affiliations:** ^1^Department of Pathology and Laboratory Medicine, Pennsylvania Hospital, Philadelphia, PA 19107, USA; ^2^Department of Pathology and Laboratory Medicine, Hospital of the University of Pennsylvania, Philadelphia, PA 19104-4280, USA; ^3^Division of Neurology, Department of Pediatrics, Children Hospital of Philadelphia, Philadelphia, PA 19104, USA

## Abstract

Connexins (Cxs) are critical for normal tissue development, differentiation, and cell proliferation. Normal expression and function of Cxs are considered to play a role in tumor suppression, but abnormal localization and abnormally increased expression of Cxs have been found in a variety of carcinomas. Of the Cx family, Cx43 is a most prevalent member and has been known as a downstream target of *β*-catenin, a key component of Wnt signaling pathway. We evaluated the expression of Cx43 in the colonic neoplasia progression sequence with additional attention to the stromal component. Resections of 50 colonic adenocarcinomas were stained immunohistochemically for Cx43 on paraffin embedded sections. Cx43 cytoplasmic expression increased progressively in the colonic adenocarcinoma sequence in both the epithelial [normal (4 ± 1), adenomatous (20 ± 2), cancerous (124 ± 10) (*P* < 0.01)], and stromal [normal (19 ± 1), cancerous (45 ± 4) (*P* < 0.01)] components. In the epithelial component, Cx43 was expressed lower in stage I adenocarcinomas (69 ± 12) compared to stage III/IV (158 ± 10, *P* < 0.01). Additionally, Cx43 was relatively increased in the adenocarcinoma at the invasive tumor front in all stages. Cx43 may play a critical role in the pathogenesis of colon cancer via gap junction or other gap junction independent mechanisms such as the Wnt/*β*-catenin pathway.

## 1. Introduction

Gap junctions are specific cell-to-cell channels formed by two hemichannels each of which is composed of six transmembrane proteins, called connexin (Cx). Gap junctions permit direct exchange of small molecules and subsequently biologic signaling between cells which are critical for tissue development, cellular differentiation, apoptosis, and cell proliferation [1, 2]. Of the Cx family, Connexin26 (Cx26), 32 (Cx32), and 43 (Cx43) are the most widely studied. While Cxs were initially thought to serve as putative tumor suppressors via normal functioning gap junctions [3–6], more recent studies have found aberrant increased expression of Cxs in a variety of carcinomas and sarcomas [7–9]. In addition, Cxs have also been observed to have transcriptional function independent of their gap junction function (36, 37). Because cell contact-mediated signaling is important in carcinogenesis by regulating invasion and metastasis [10–12], we hypothesize that Cxs may also serve a role in these processes through gap junction dependent or independent mechanisms. Additionally, because epithelial stromal interactions and signaling are also critical for normal cell biology and cancer cell migration, we hypothesized that the expression of Cxs may be altered in the mesenchymal tissue in neoplastic progression. 

Colon cancer is one of the most common cancers in the Western countries and serves as an ideal model system to study neoplastic progression. Specifically, resection specimens usually contain normal colon, the adenomatous precursor lesion, variably invasive cancer, and, depending on stage, metastatic disease. Cx43, an important member of the Cx family, has been shown to be present in the normal human epithelium of the colon [13, 14]. However, the role of Cx43 in the physiology of the colon is currently poorly understood. Aberrant Cx43 expression has been found in several types of tumor, including liver, prostate, breast, and lung [12, 15–21]. Importantly, *GJA1*/Cx43 has been reported to be frequently mutated in tumors of the colon, suggesting that inactivation of Cx43 can be involved in colorectal carcinogenesis [14]. However, the role of Cx43 in carcinogenesis remains to be elucidated. 

Adenomatous polyposis coli (APC) plays important roles in a wide range of cellular functions such as proliferation, migration, differentiation, and apoptosis in colonocytes [22–25]. APC is important in Wnt signalling, where it participates together with axin and glycogen synthase kinase 3b (GSK3b) to target the adhesion molecule and transcription factor *β*-catenin for degradation. APC mutations have been found in 40–80% of sporadic colon cancer and in almost all cases of familial adenomatous polyposis [26–28]. The truncated APC gene product causes dysregulation of *β*-catenin. When van der Heyden et al. [29] investigated the effects of Wnt1 overexpression on gap junctional communication in PC12 cells, they reported that Wnt1 expressing clones displayed an increased electrical and chemical coupling. This coincided with an increased expression of Cx43 mRNA. Also, induction of Wnt1 expression in a mammary epithelial cell line leads to an increase in gap junctional communication and Cx43 protein expression. In the absence of functional APC, *β*-catenin accumulates in the nucleus where it can turn on transcription of several genes, including the gap junction protein Cx43, COX-2, cyclin-D1, and PPAR*δ* [29–33]. Therefore, Cx43 could be a potential biomarker for colorectal carcinoma.

To the best of our knowledge, the importance of connexin43 in colorectal carcinogenesis has not been well investigated. Little is known about the expression of Cx43 in colorectal carcinoma; especially in clinically and pathologically well-characterized cases, the goal of our study was to evaluate the expression of Cx43 in a series of well characterized colorectal adenocarcinomas. Specifically, we studied (1) the expression and localization of Cx43 in colon cancer progression sequence with attention to both epithelial and stromal compartments and (2) correlation of Cx43 expression in colon cancer with its pathologic stage and histologic grade. 

## 2. Materials and Methods

This retrospective study was performed in 50 cases of primary resections of colonic adenocarcinoma between the years 2000 to 2005 from the Hospital of the University of Pennsylvania. It was approved by the Institutional Review Boards (IRB). Cases with macroscopic or microscopic residue of tumor cells at the surgical margins and those with preoperative chemo- or irradiation therapy were excluded. All cases were histopathologically diagnosed according to the American Joint Committee on Cancer (AJCC) classification and TNM staging. Lymph node metastases were checked by histopathological examination in all cases. Distant metastases were diagnosed by histopathological examination. A representative block was selected in each case for the study. Normal colonic mucosa was present in the selected block in 37 cases and adenoma in 14 cases.

### 2.1. Immunohistochemistry

Tissue specimens were fixed in 10% formalin and embedded in paraffin in all cases. Sections (5 *μ*m) were deparaffinized with Xylene 3 times for 3–5 minutes each. Antigen retrieval was performed in 10 mmoL/L of sodium citrate (pH 7.6) in a microwave for 4 minutes twice at 70% power level. Endogenous peroxidase was inactivated by incubation in 5% hydrogen peroxide for 5 minutes. Nonspecific binding sites were blocked by incubating with 2% normal horse serum for 20 minutes. Connexin43 immunohistochemical stains were performed with a polyclonal goat connexin43 antibody (clone CXN-6, Santa Cruz Biotechnology) with 1 : 200 dilution and incubation at room temperature for 60 minutes. Immunoreactivity was visualized by using EnVision+ system—HRP labeled polymer on a DAKO autostainer (DAKO, Carpinteria, CA).

**Double Cx43 and Beta-Catenin Staining**

Cx43 stain was performed as above said. Afterwards the beta-catenin stain was performed manually. After boiled in 1× citrate buffer for 20 minutes, sections were incubated with mouse anti-beta-catenin antibody (monoclonal, BD Bioscience cat no. 610154) at 1 : 250 dilution for 30 minutes at room temperature followed by alkaline phosphatase-conjugated goat antimouse antibody (DAKO, Carpinteria, CA) at 1 : 75 dilution for 30 minutes and then exposed to Perma Red (Dako, Carpinteria, CA) for 5 minutes at RT and counterstained with hematoxylin.

### 2.2. Evaluation of Cx43 Immunostaining

Cx43 immunostaining was evaluated by three authors independently, blinded to patient outcome and all clinicopathologic findings. The immunohistochemical staining was analyzed and classified into four groups based on the staining intensity (0, absent; 1+, weak; 2+, intermediate; 3+, strong staining). The percentage (%) and staining intensity (0–3) of epithelial and stromal cells in normal, adenomatous, and cancerous areas were determined. The total staining score was calculated by the sum of staining intensity multiplied by its percentage yielding a possible score of 0–300. In the case of heterogeneous staining within the same sample, we determined the percentage of different staining intensities individually in each area and calculated the total sum.

### 2.3. Statistical Analysis

Statistical analysis was done using the Stata software (StataCorp, College Station, TX). The association of staining intensity with clinicopathologic patterns was assessed with unpaired Student *t*-test, when appropriate. All data were expressed as the mean ± SR. *P* values of < 0.05 were accepted as statistically significant.

## 3. Results

### 3.1. Clinicopathological Characteristics of Patients with Colon Cancer

The average age of the patients was 61 years (range, 35–82; SD, 14.28 years). Of 50 patients, 19 (38%) were diagnosed as AJCC TNM stage I, 22 (44%) were diagnosed as stage III, and 9 (18%) were diagnosed as stage IV. Histological grades of the tumor are low in 2 (4%), moderate in 36 (72%), and high in 12 (24%) cases. There is no significant difference in age and sex between stage I and stage III/IV (Table 1). Although more cases with poorly differentiated adenocarcinoma in stage III/IV are seen, there is no histologic grade difference between stage I and stage III/IV (Table 1).

### 3.2. Expression of Cx43 in the Neoplastic Epithelium (Colon Cancer Progression Pathway)

**Figure 1:**
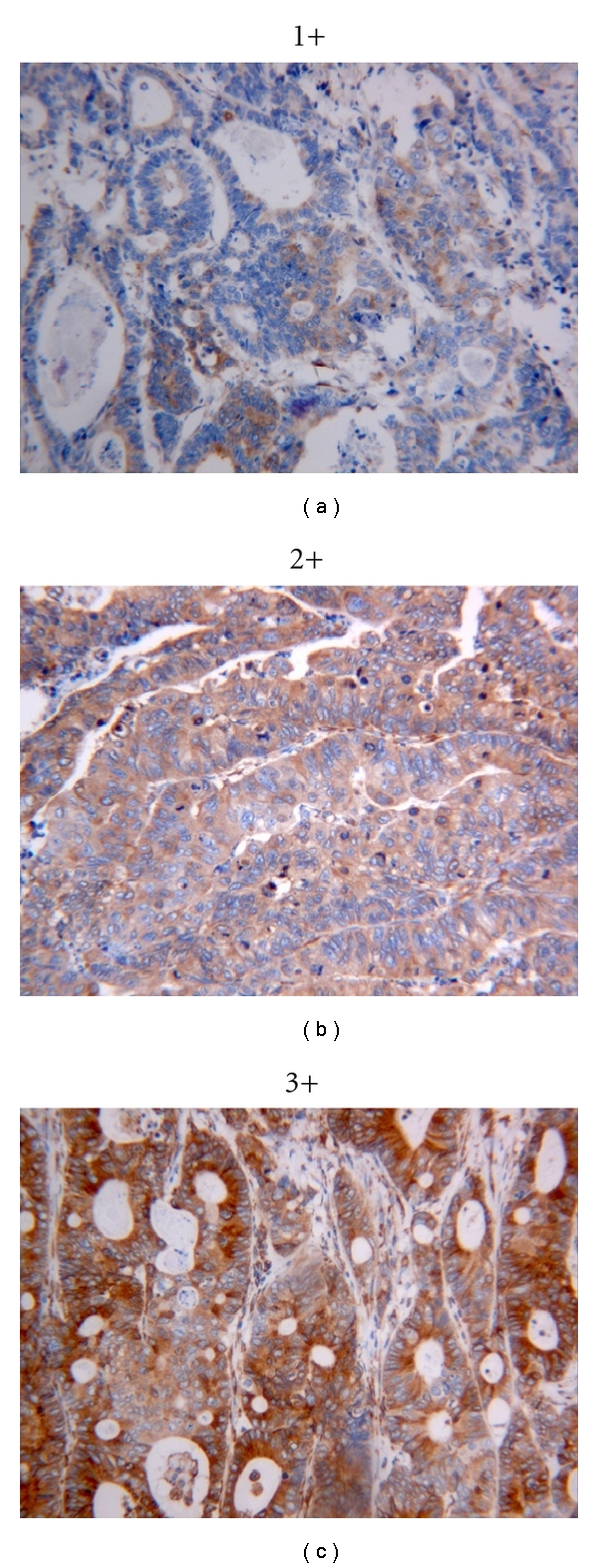
The expression of Cx43 in colonic adenocarcinoma was evaluated according to the intensity of the staining as follows: 1, very weak expression (1+); 2, moderate expression (2+); 3, strong expression (3+) (Original magnification ×200).

**Figure 2:**
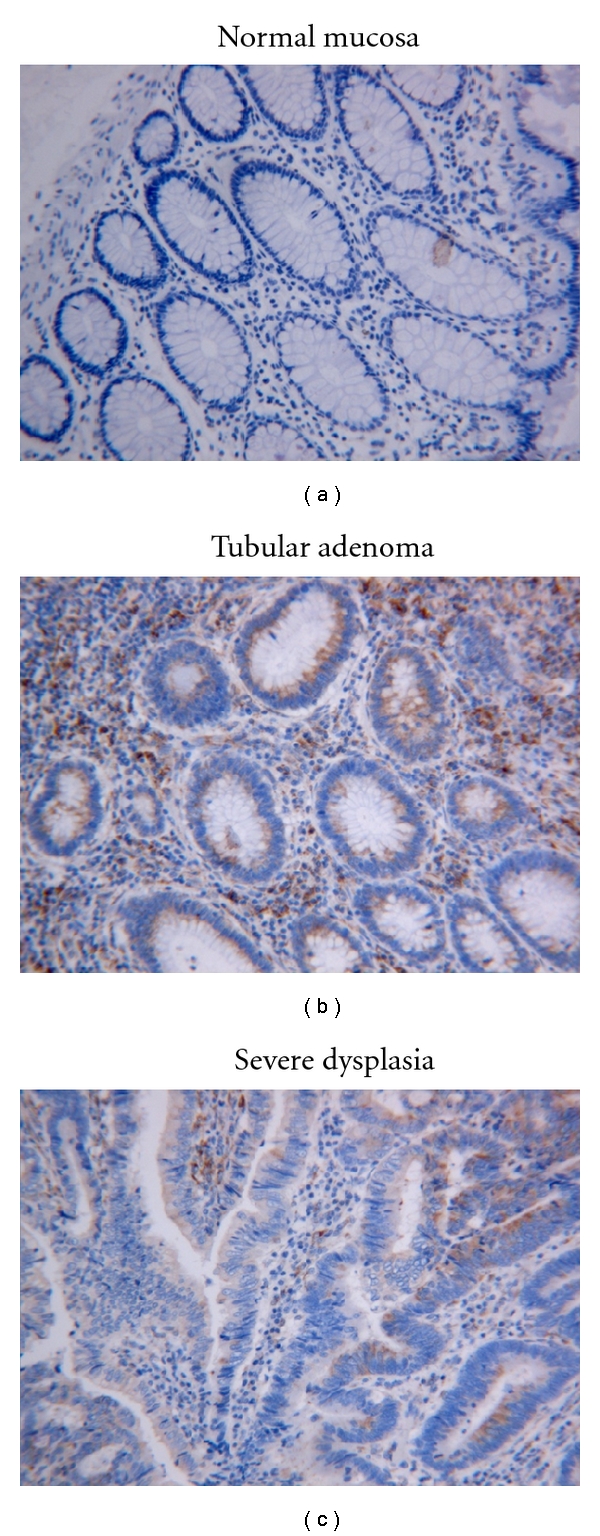
The expression of Cx43 in normal colonic mucosa, tubular adenoma, and severe dysplasia (Original magnification ×200). There is an increase in cytoplasmic Cx43 expression from normal epithelium (Cx43 score 4 ± 1) to tubular adenoma/severe dysplasia (Cx43 score 20 ± 2).

**Figure 3:**
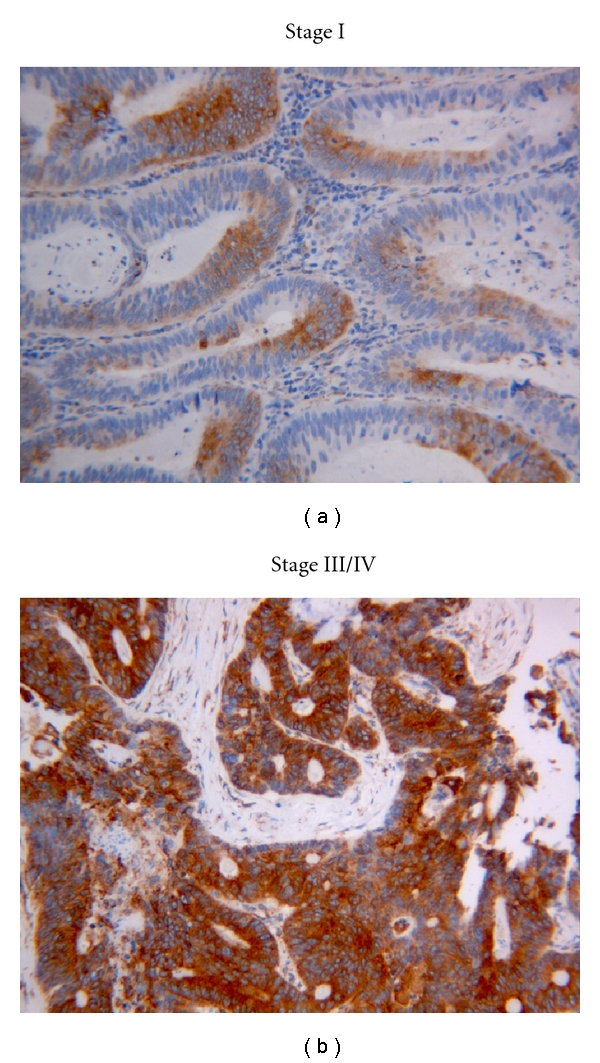
The difference in Cx43 expression in stage I and stage III/IV colonic adenocarcinoma (original magnification ×200). A higher level of Cx43 expression was significantly associated with AJCC stage III/IV adenocarcinomas (Cx43 score 158 ± 10), as compared to AJCC stage I (Cx43 score 69 ± 12).

Figure 1 depicts the different Cx43 staining intensity in colorectal adenocarcinoma. Although Cx43 protein was reported to express in normal colonic mucosa [14], we only rarely observed punctuate intercellular staining of Cx43 in normal colonic mucosa. In contrast, we found a progressive increase in cytoplasmic Cx43 expression from normal epithelium to tubular adenoma/severe dysplasia (Figure 2) and to carcinoma (Figure 3). Cx43 score according to each histologic category was recorded as 4 ± 1 in normal (*n* = 37), 20 ± 2 in adenoma (*n* = 14), and 124 ± 10 in cancer (*n* = 50) (*P* < 0.01). Variable Cx43 expression was detected in colon cancer tissues in all cases (50/50). Cx43 was expressed predominantly in the cytoplasm in colon cancer and precancer epithelia, although a mixed (cytoplasmic and membranous) staining in stage III/IV colonic carcinoma was seen. Additionally, Cx43 reactivity was relatively increased in the invasive front of the adenocarcinoma in all cases of different stages (data not shown).

### 3.3. Expression of Cx43 in Stromal Component

**Figure 4:**
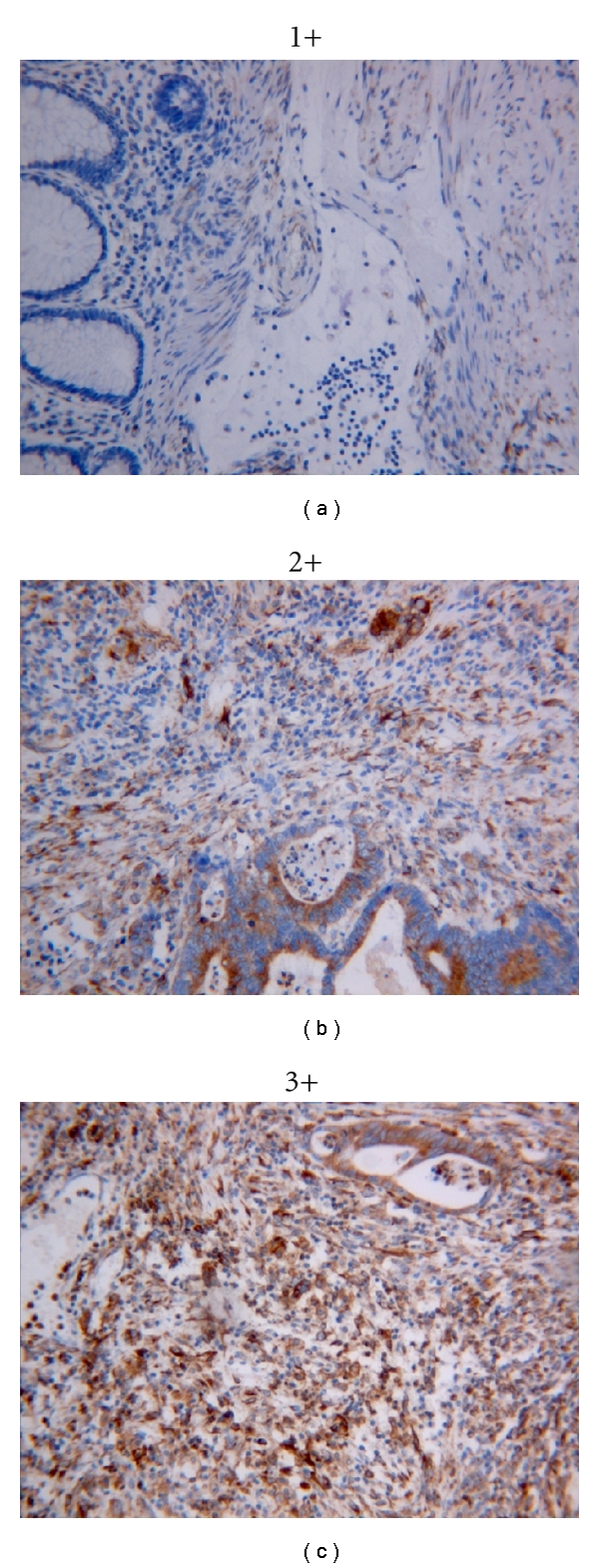
The expression of Cx43 in colonic stroma was evaluated according to the intensity of the staining as follows: 1, very weak expression (1+); 2, moderate expression (2+); 3, strong expression (3+) (original magnification ×200).

**Figure 6:**
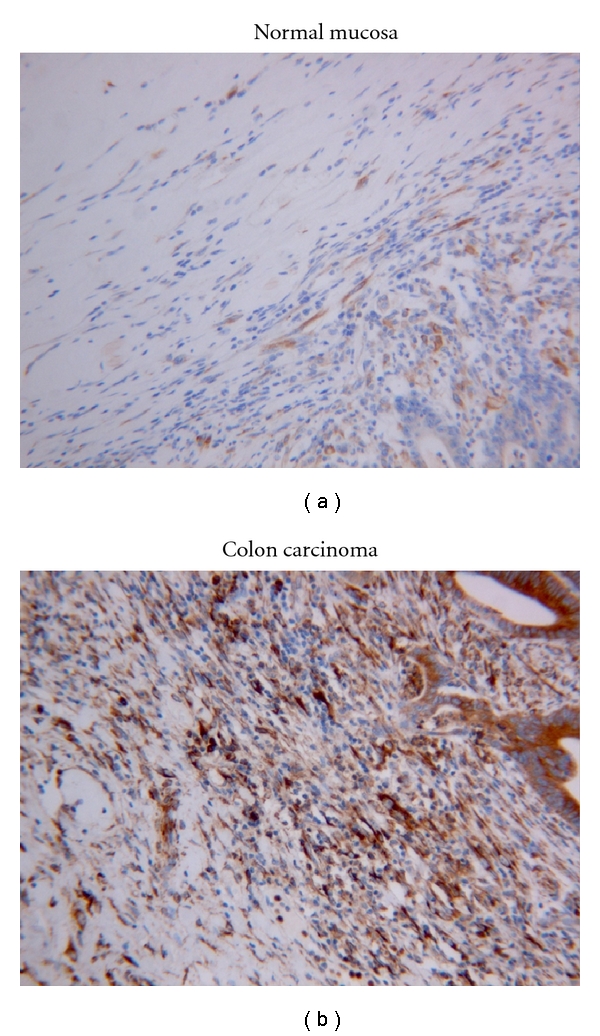
The difference in Cx43 expression in the stromal component in normal mucosa and colonic adenocarcinoma (original magnification ×200). Cx43 reactivity was stronger in the stromal components adjacent to cancerous epithelium than benign epithelium: normal (Cx43 score 19 ± 1), cancer (Cx43 score 45 ± 4).

Cx43 expression was also localized to connective tissue and muscular tissue in the samples, primarily in the connective tissue in close proximity to epithelial cells (Figure 4). Cx43 reactivity was stronger in the stromal components adjacent to cancerous epithelium than benign epithelium: normal (Cx43 score 19 ± 1), cancer (Cx43 score 45 ± 4) (*P* < 0.01) (Figure 6). However, there is no significant stage difference (Stage I: 42 ± 4, Stage III/IV: 46 ± 6) (*P* > 0.05).

### 3.4. Expression of Cx43 in Colonic Adenocarcinoma in Relation to Stage and Grade

A higher level of Cx43 expression was significantly associated with AJCC stage III/IV adenocarcinomas (158 ± 10), (*P* < 0.01) as compared to AJCC stage I (69 ± 12) (Figure 3). Cx43 expression did not show any correlation with histologic grade.

### 3.5. Coexpression of Cx43 and Beta-Catenin

**Figure 5:**
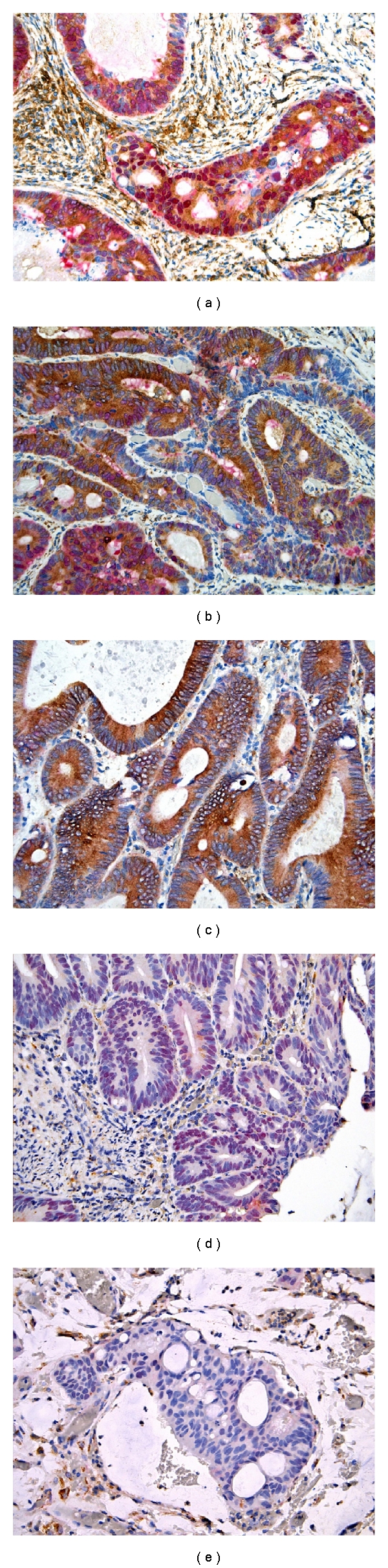
The relationship between Cx43 expression and nuclear beta-catenin expression in colonic adenocarcinoma (original magnification ×200). (a) Coexpression of Cx43 (brown cytoplasmic staining) and nuclear beta-catenin (red nuclear staining). *Note.* Peritumoral stromal cells exhibited only Cx43 but not beta-catenin reactivity. (b) Tumoral tissue exhibited heterogeneous expression of Cx43 and nuclear beta-catenin. (c) Tumoral tissue with expression of Cx43 only. (d) Tumoral tissue with expression of beta-catenin only. (e) Tumoral tissue with no expression of Cx43 or beta-catenin.

After evaluation of the initial Cx43 staining, 12 cases, 6 with low Cx43 reactivity and 6 with high Cx43 reactivity, were selected for double staining. Nuclear and sometimes cytoplasmic beta-catenin reactivity (red) was observed in neoplastic cells. However, for practical reason, only nuclear reactivity was analyzed against the Cx43 cytoplasmic reactivity (brown) on double staining. No nuclear beta-catenin reactivity was detected in nonneoplastic colonic epithelium. Coexpression of Cx43 and beta-catenin was commonly seen but is not exclusive as other tumor cells in the same tumor frequently exhibited only either Cx43 or beta-catenin. There was no correlation in the level of immunoreactivity between Cx43 and beta-catenin (data not shown). No nuclear beta-catenin reactivity was detected in any stromal cells regardless of their Cx43 reactivity (Figure 5).

## 4. Discussion

It was suggested that loss of gap junction protein expression, aberrant cytoplasmic localization, and disturbance of gap junction intercellular communication would be important events in carcinogenesis, invasion, and metastasis [12, 34]. There has been a large body of literature to suggest that gap junctions are implicated in cellular growth control and tissue differentiation, and normal membranous expression of Cxs has tumor suppressive effect that controls tumor progression by regulating cell growth and differentiation. However, the role of Cxs in carcinogenesis and metastasis remains controversial, because it is still unclear whether Cx expression is required for invasion and metastasizing [35]. 

In our study, the intercellular/membranous staining was rare and only sporadically seen in normal colonic epithelium, but cytoplasmic expression of Cx43 was frequently observed in colonic cancer cells. In addition, we found that the level of Cx43 expression was significantly associated with higher AJCC stage III/IV but did not have impact on the histological grading. We speculate that cytoplasmic expression of Cx43 might reflect transcriptional or posttranscriptional defects of this protein during colorectal carcinogenesis, or a product of the mutated gene, a common event in colorectal cancer [14]. If connexins do not assemble to form functional gap junction channels such as in the case of aberrant cytoplasmic accumulation and expression, they might cause alteration in expression of different genes in cooperation with other proteins [36, 37], or they may function as adhesion proteins to form adhesive plaques that could severely impair signaling pathways [38]. It has been demonstrated that loss of intercellular communication correlates with high metastatic potential of mammary adenocarcinoma cells [18] and that connexins may be involved in intravasation and extravasation of lung cancerous cells [12]. It has been speculated that connexins may play an important role in the extravasation of cancerous cells into lymphoid tissue by formation of gap junctions between tumor cells and endothelial cells in lymph node vessels [18]. Increased expression of Cx43 has been reported in lymph node metastases compared to primary tumor to suggest a potential role of Cx43 in metastatic process [18, 39]. Dubina et al. [14] reported that the mutational alterations in the carboxyl-terminal region of Cx43 are involved in advanced stages of progression of human colon cancer. The carboxyl-terminal tail of Cx43 contains several motifs for phosphorylation by different protein kinases, which is essential for trafficking, gap junction assembly, channel gating, and turnover of Cx43. The frameshift mutations in colon tumors appear to abolish particularly effectively most if not all functional properties of this part of the protein and thus could have a strong impact on cell growth, morphology, and motility that would be critical for cancer progression. However, the fact that Cx43 mutations have been found only in invasive structures of exophytic colon adenocarcinoma, but neither among their benign precursors adenomatous polyps nor in endophytic carcinomas, suggests that Cx43 mutational alterations are limited to this specific growth type of human colon neoplasms and are involved at a fairly late stage of their progression [14]. Nevertheless, more and more evidences have merged to suggest that the loss of normal function of connexins during carcinogenesis is not only due to gene mutations, but, more importantly, also due to the multiple steps of possible alteration in connexin expression, including lack of transcription of connexin genes, lack of translation of connexin mRNA, and lack of membrane targeting leading to accumulation of connexin proteins in the cytoplasm. As Cx43 is a target for *β*-catenin/Tcf-mediated transcription [29], accumulated *β*-catenin in colon cancer may increase Cx43 expression in colonic mucosal epithelium. This hypothesis was supported by the finding in cardiomyocytes in which *β*-catenin was shown to interact directly with Cx43 and to transactivate Cx43 [40]. Our study of double staining revealed that colorectal adenocarcinomas commonly expressed both beta-catenin and Cx43 in the same or different cells but failed to demonstrate correlation of expression of these two factors in situ. However, these results were not completely unexpected given the fact that beta-catenin immunoreactivity has not been consistently shown to correlate with various pathologic and clinical parameters of colorectal cancer in literatures [41, 42]. The role of beta-catenin in regulating Cx43 in colorectal cancer is likely more complex than expected and needs to be further evaluated. The lack of any nuclear beta-catenin reactivity seen in the Cx43 positive peritumoral stromal cells also suggested other pathways involved in Cx43 expression in colorectal cancer. In any case, our findings of higher Cx43 expression in neoplastic epithelium and advanced stage of colorectal cancer are consistent with these previous observations on the role of Cx43 in clinically well-characterized cases and suggest an oncogenic role of Cx43 which might be gap junction dependent or independent. 

Cx43 expression was also located to connective tissue and muscular tissue in benign bowel and colon cancer. We observed that Cx43 was relatively elevated in the connective tissue in close proximity to epithelial cells, and higher Cx43 reactivity was in the stromal components adjacent to cancer than to benign epithelium. It is thought that the role of gap junctions in epithelial-stromal interactions may be important in carcinogenesis. Husøy et al. [30] found an increased expression of Cx43 in the stroma around intestinal tumors in animal (mice) with the multiple intestinal neoplasia (Min). Furthermore, the increased Cx43 expression in stromal myofibroblasts was colocalized with COX-2, which expression is well known to be increased in both humans and mice adenomas. Myofibroblasts are suggested to secrete signaling molecules that could stimulate invasion of cancer cells, since primary cultures of subepithelial myofibroblasts from human colon promote the migration of epithelial cells [43]. Similar to adenomas in Min mouse intestine, Cx43 has also been reported to be increased in stromal cells of human breast carcinomas. Therefore, it is conceivable that peritumoral stromal Cx43 expression observed in our study might reflect a similar biologic function of Cx43 in colonic carcinogenesis as observed in the animal and experimental models. The significance of increased peritumoral stromal Cx43 expression in carcinogenesis needs to be further investigated for its role in epithelial-mesenchymal transition/interaction.

In conclusion, in this study, we have found that the level of Cx43 expression progressively increased along the colonic carcinoma progression sequence and advanced tumor stage in the neoplastic epithelium and the stroma surrounding cancerous epithelium. The findings suggest that connexin43 may play a critical role in the pathogenesis of colon cancer likely via multifactorial mechanisms including, but not limited to, abnormal gap junction formation, transcriptional activity of cytoplasmic Cx43, and abnormal epithelial-stromal interactions. Study is needed to further understand the molecular basis of Cx43 oncologic effect in colon cancer and how Cx43 is regulated in colonic carcinoma progression sequence. Immunohistochemical evaluation of Cx43 expression might have prognostic significance as there is a correlation of significantly higher level of Cx43 expression with more advanced stage colon carcinomas. However, evaluation of Cx43 in larger cohort of cases with clinical outcome data is needed to further assess this potential prognostic role of Cx43 in colorectal cancer.

## Figures and Tables

**Table 1:** Clinicopathological characteristics of patients with colon cancer.

Parameters	Stage I (*N* = 19)	Stag III/IV (*N* = 31)	*P*
Median age	60.0 ± 14.0	61.5 ± 10.5	>0.05
Male : female ratio	8 : 11	18 : 13	>0.05
Histologic grade			
WD	2	0	
MD	15	21	
PD	2	10	>0.05

WD: Well differentiated, MD: Moderately differentiated, PD: Poorly differentiate.
